# Hematological profiles of newborns of mothers with hypertensive disorders of pregnancy delivered at the University of Gondar comprehensive specialized hospital: a comparative cross-sectional study

**DOI:** 10.1186/s12887-023-04491-3

**Published:** 2024-01-05

**Authors:** Zewudu Mulatie, Melak Aynalem, Solomon Getawa

**Affiliations:** 1 Department of Medical Laboratory Sciences, College of Medicine and Health Sciences, Wollo University, Dessie, Ethiopia; 2 Department of Hematology and Immunohematology, School of Biomedical and Laboratory Sciences, College of Medicine and Health Sciences, University of Gondar, Gondar, Ethiopia

**Keywords:** Hematological profile, Hypertensive disorder, Newborns, Gondar, Ethiopia

## Abstract

**Background:**

Hypertensive disorders in pregnancy can cause prenatal placental perfusion with insufficient blood supply to the fetus, resulting in fetal exposure to hypoxia and leading to disturbance of neonatal hematopoietic stem cells. This study aimed to compare the hematological profiles of newborns from mothers with hypertensive disorders and normotensive delivered at the University of Gondar comprehensive specialized hospital.

**Methods:**

A comparative cross-sectional study was conducted from March to May 2022 among 308 newborns from hypertensive and normotensive mothers in equal proportions. A systematic random sampling technique was used to select study participants. Three milliliters of cord blood were collected to perform a complete blood count by Beckman coulter. The results were presented using tables and graphs. Independent t-test and Mann-Whitney U test were done to compare the hematological profiles of the two groups. P-value < 0.05were considered statistically significant.

**Results:**

The majority of hypertensive and normotensive mothers’ ages were between 20 and 34 years (83.77% and 90.91%, respectively). The hematocrit levels were significantly higher in neonates of hypertensive mothers than the neonates of normotensive mothers (49.10 ± 5.19% and 46.09 ± 7.63% respectively) (P < 0.001) while neutrophil counts were significantly lower in neonates of hypertensive mothers than the neonates of normotensive mothers (6.62 ± 3.30 and 7.55 ± 3.31 × 10^3^ /ul respectively) (P = 0.007). Also, platelets counts were significantly lower in neonates of hypertensive mothers than neonates of normotensive mothers (221.25 ± 83.56 and 260.24 ± 83.01 × 10^3^/ul respectively) (P < 0.001). The platelet and nucleated red blood cell count showed a statistically significant difference among newborns from mothers with superimposed preeclampsia and gestational hypertension.

**Conclusion:**

Newborns delivered from hypertensive disorders of pregnancy had low white blood cell parameters, low platelet count and high red blood cell parameters compared to controls. As result, newborns may develop leukopenia, thrombocytopenia and polycythemia, respectively. Therefore, newborns should be monitored for early detection and follow-up of hematological abnormalities before complications occurred.

## Background

Hypertensive Disorder of Pregnancy (HDP) is a hypertensive disorder that occurs before or during the pregnancy period [1, 2]. The American College of Obstetricians and Gynaecologists classified HDP into chronic hypertension, gestational hypertension, preeclampsia/eclampsia, and superimposed preeclampsia/eclampsia [3].

Globally, HDP is the main cause of maternal and neonatal morbidity and mortality [4]. It is responsible for a 10% prenatal and neonatal death rate, as well as 40–50% of low birth weight babies [5]. According to the World Health Organization study, HDP accounts for 14% of maternal mortality in sub-Saharan Africa and is the second leading cause of maternal death after hemorrhage, which accounts for 16.0% of maternal mortality [6]. Approximately 6.07% of pregnant women in Ethiopia develop HDP [7].

Hypertensive disorders in pregnancy result in a decline in prenatal placental perfusion with insufficient blood supply to the fetus, resulting in fetal exposure to hypoxia or oxidative stress in the placenta. In response to this stress, the hypoxic placenta releases certain vasoactive substances into the mother’s blood, which alters the endothelium layer’s permeability and alters the vascular response to this stress [8, 9]. Reduced oxygen tension triggers compensatory mechanisms that increase erythrocytes as well as an increase in erythroblasts and nucleated red blood cell (nRBC) numbers [10, 11]. Previous studies showed that newborns from hypertensive mothers had a higher rate of polycythemia than newborns from normotensive mothers [12, 13].

Hypertensive disorders in pregnancy cause neonatal neutropenia as a result, increasing the risk of nosocomial and other infections, and it has also been shown to predict sepsis independently [14]. The possible mechanism of the HDP causing neutropenia is that the resulting uteroplacental insufficiency inhibits fetal myeloid bone marrow production, resulting in a decrease in neutrophil count [15].

Hypertensive disorders in pregnancy and their fetal hypoxia are thought to have a direct depressant effect on fetal megakaryocytopoiesis and platelet production [11]. Newborns born to hypertensive mothers are more susceptible to bleeding disorders than newborns delivered to normotensive mothers [15, 16]. Neonatal thrombocytopenia affects 36% of babies born to mothers who have hypertension, which is particularly common in premature and low birth weight babies [17].

Maternal hypertension has a direct impact on the development of hematological abnormalities in newborns, whereas premature delivery and weight of the newborns are additional aggravating factors that affect the hematological profiles of newborns [18]. Other factors that affect the hematological profiles of newborns are increased blood pressure, and increasing the fetus’s exposure to hypertension for a long time has a great impact on the hematological profiles of newborns [19].

Studies were conducted in developed and developing countries about the effect of HDP on the hematological profiles of newborns, but the results have been inconsistent, especially for white blood cell and red blood cell parameters due to variations in ethnicity, nutrition, genetics, and environmental factors [20]. There is also a scarcity of data in Ethiopia regarding the hematological profiles of newborns from mothers with HDP. Therefore, the study aimed to compare the hematological profiles of newborns from mothers with HDP and normotensive mothers delivered at the University of Gondar comprehensive specialized hospital.

## Materials and methods

### Study design, area, and period

A comparative cross-sectional study was conducted at the University of Gondar comprehensive specialized hospital from March to May 2022. The hospital is located in Gondar town. It is located 738 km away from Addis Ababa, and 180 km away from Bahir Dar, the capital city of the Amhara region. The town is located at latitude 12^o^361N and longitude 37^o^281E. Also, it is located at an elevation of 2133 m above sea level. The hospital has several wards and outpatient sections; the hospital provides various medical services to more than 7 million people in the zones and individuals from neighbouring zones. The hospital has more than 550 beds, and it handles approximately 10,000 newborns per year. The delivery ward of the hospital has 4 rooms and 54 beds [21].

### Populations

The source populations for cases were all newborns delivered from mothers with hypertensive disorders at the University of Gondar comprehensive specialized hospital during the study period. Also, the source populations for controls were all newborns delivered from normotensive mothers at the University of Gondar comprehensive specialized hospital during study period. All newborns delivered from mothers with HDP and normotensive mothers at the University of Gondar comprehensive specialized hospital during the study period who fulfilled the inclusion criteria were taken as the study population for the case and control groups, respectively.

### Inclusion and exclusion criteria

Newborns from mothers who had SBP ≥ 140 mmHg or DBP ≥ 90 mmHg, or both on more than one occasion at least 4–6 h apart were included in the case. Newborns from normotensive mothers who had SBP between 90 and 120 mmHg and DBP between 60 and 80 mmHg were also included as controls. The exclusion criteria for both newborns of hypertensive and normotensive mothers were those with a history of conditions such as severe anemia, diabetes mellitus, heart failure, renal failure, liver disease, and febrile illnesses that could alter their newborns’ hematological profiles. Additionally, the newborns of HIV/AIDS positive mothers [22], the newborns of mothers who had taken drugs like aspirin and warfarin [23], mothers who had malaria during pregnancy and a history of blood transfusion within the last 3 months during pregnancy that may change the hematological profiles of newborns [24]. So, these participants were excluded from the study. Furthermore, the newborns who were born in a twin pregnancy, and newborns with ABO/Rh incompatibility, congenital anomalies, severe birth asphyxia, and stillbirths were excluded from the study. Those medical conditions were excluded by using a medical chart and screening tests done during data collection time.

### Sampling size determination and sampling technique

The sample size was calculated using a standard formula for estimating double population means for newborns from hypertensive and normotensive mothers by using open Epi version 3, taking 95% confidence level, 80% power, the ratio is one, and the mean of the hematocrit for newborns from hypertensive mothers was 48.4 ± 9.1, and for newborns from normotensive mothers was 45.8 ± 7.0 in Nigeria [11], because there is no study conducted in Ethiopia or in the study area. All parameters were checked when showed statistically significant for sample determination. But the sample sizes were lower when other parameters were used for sample determination. The total sample size was 308. Therefore, 154 newborns were taken for each group.

A systematic random sampling technique was used to select cases and controls attending the University of Gondar comprehensive specialized hospital. The samples were selected by preparing the sample frame for cases and controls. This was done by knowing the total number of newborns from hypertensive mothers during the study period and the sample size of newborns from hypertensive mothers. The total number of newborns from HDP mothers delivered at the hospital per day was approximately 6. So, the total number of cases in three months was 540. The K value of the cases was calculated as K = N/n = 540/154 = 3.50. Therefore, the samples of the case were selected at every 3 intervals. Also, for control, the total number of newborns attended to at the hospital per day was approximately 10. The interval for selecting the samples of controls was K = 810/154 = 5.25. Therefore, the samples were selected every 5 intervals. The first sample was selected by lottery methods. The excluded study participants were substituted with the next consecutive study participants.

### Operational definitions

#### Hypertensive mother

Is the mother SBP ≥ 140mmHg or DBP ≥ 90mmHg or both on more than one occasion at least 4–6 h apart [1, 2].

#### Normotensive mother

Is mother SBP between 90 and 120 mmHg and DBP between 60 and 80 mmHg [1, 2].

#### Chronic Hypertension

the mothers develops high blood pressure before pregnancy or is detected within the first 20 weeks of pregnancy, or does not improve by the 12-week postpartum checkup [25].

#### Gestational Hypertension

The mothers develop a blood pressure of ≥ 140/90 mmHg on two occasions (at least 4 h apart) after 20 weeks’ gestation in a previously normotensive woman, without the presence of proteinuria or other clinical features (thrombocytopenia, impaired renal or kidney function, pulmonary edema, or new-onset headache) [26].

#### Superimposed preeclampsia/ eclampsia

The mothers develops hypertension before 20 weeks of pregnancy, but proteinuria is detected after 20 weeks of pregnancy [27].

#### Preeclampsia

The blood pressure of mothers exceeds 140/90 mmHg occurred after 20 weeks of pregnancy and other clinical features (thrombocytopenia, impaired renal or kidney function and pulmonary edema) [28].

#### Thrombocytopenia

The platelet count of newborns is less than 132.7 × 10^9^ /L [29].

#### Anemia

The level of Hgb of newborns is less than 13.3 g/dl [29].

#### Polycythemia

The level of hematocrit of newborns is greater than 58.1% [29].

#### Leukopenia

The TLC of newborns is less than 7.64 × 10^9^ /L [29].

#### Neutropenia

The ANC of newborns is less than 2.96 × 10^9^ /L [29].

#### Very low birth weight

The weights of the newborn were less than 1500 g [30].

#### Low birth weight

The weights of the newborn were between 1500 and 2500 g [30].

#### Normal birth weight

The weights of the newborn were from 2500 to 4000 g [30].

#### High birth weight

the weights of the newborn were greater than 4000 g [30].

#### Pre term

Babies born alive within 37 weeks of pregnancy are completed [30].

#### Full term

Babies born after 37 weeks of gestation are completed [30].

#### Clumping time

The cord is clamped before 5 min [31].

#### Primigravida

A woman who becomes pregnant for the first time [32].

#### Multigravida

A woman who becomes pregnant for more than one time [33].

Maternal malnutrition: A mother lack of sufficient food or the deficiency of a specific nutrient, such as iodine, iron, folate, calcium and zinc [34].

### Data collection and laboratory methods

#### Socio-demographic and clinical data collection

A pre-tested structured questionnaire prepared in English and translated to the local language (Amharic) was used to collect the maternal socio-demographics, the nutritional status of mothers, and the sex of newborns via face-to-face interviews of mothers/guardians. The clinical characteristics were collected from the mother’s medical charts by using a data extraction checklist. Additionally, the mothers came for only delivery service in the Hospital, so the clinical characteristics of mothers were done before delivery. The weight and the height of mothers were measured before delivery. Also, the weight of newborns was measured after delivery by beam balance. The data and cord blood specimens was collected by trained midwives and/or nurses.

#### Blood specimen collection and processing

After delivery, approximately 3 milliliters of umbilical cord blood specimens were obtained from the clamped umbilical cord. The umbilical cord was clamped immediately and cleaned with 70% alcohol and an iodine swab to remove maternal blood and contaminants. After cleaning, the cord blood sample was collected by the syringe method. Then the sample was transferred into a test tube containing di potassium Ethylene Diamine Tetra Acetate (EDTA) and gently mixed to prevent clotting. The cord blood specimen was transported to the hematology laboratory for a complete blood count (CBC) analysis. Also, blood samples were collected from mothers who came for only delivery service in the hospital for hematological, serological, and chemistry tests. The laboratory tests were performed in the clinical laboratory of University of Gondar Comprehensive Specialized Hospital, and the tests performed by laboratory technologists who work in the clinical laboratory.

#### Data quality control

A pre-test was done to assess the integrity of the questionnaire on 5% of the sample size at Maraki health center in Gondar town before the actual data collection process began. Based on the feedback, an amendment was made to ensure precision and reliability. The quality of the data was achieved by using operational definition, training data collectors for one day, and supervising the data collection process. The cord blood samples were collected, prepared, and tested according to standard operating procedure (SOP) to get a reliable result from the study. The samples were checked to see if they met acceptable criteria like hemolysis, clotting, volume, collection time, and correct labelling. The Beckman Coulter UniCel® DxH 800 hematology analyzer was checked by using commercially prepared 6 C cell quality control reagents.

#### Data processing and data analysis

The hematological tests were carried out using a Beckman Coulter hematology analyzer model Unicel DxH 800. Beckman Coulter utilized different principles for the measurement of the hematology parameters. The volumetric impedance method was used for determining the WBC, RBC, and PLT count. The optical light scatter and diffraction method was used to determine the 5-part WBC differential. Another method is the photometric light absorbance which used for determining the Hgb. In addition the reticulocyte count based on super vital staining methods. The data was entered and cleaned by using Epidata version 3.1 software. After checking the data quality, the data was transferred into Stata 14 software and used for statistical analysis. The results were presented using tables and graphs. Also, descriptive statistics such as percentage, mean, median, interquartile range (IQR), and standard deviation (SD) were calculated. The data were checked for normality by the Shapiro-Wilk test. An independent sample t-test and a Mann-Whitney U test were used for the comparison of hematological profiles between cases and controls for the normal distribution and skewed data, respectively. Additionally, an Analysis of variance (ANOVA) test for normally distributed data and a Kruskal-Wallis test for skewed data were used for the comparison of hematological profiles of newborns from mothers with different types of HDP. A Bonferroni post hoc test was used to identify the hematological profiles of newborns between different types of HDP that showed significant differences. Pearson correlation was used to determine the correlation between the hematological profiles of newborns and independent variables when the data had a normal distribution. However, Spearman’s rank correlation was used when the data were not normally distributed. The P-value < 0.05 was considered statistically significant.

## Results

### Socio-demographic characteristics of newborns and mothers

In this study, a total of 308 newborns (154 cases and 154 controls) were involved. The sex of newborns being female from hypertensive mothers was 58.44%, while from normotensive mothers it was 54.55%. The majority of the hypertensive and normotensive mothers’ ages were between 20 and 34 years (83.77% and 90.91%, respectively). Out of 154 hypertensive mothers, 61.04% were urban dwellers, while out of 154 normotensive mothers, 72.73% were urban dwellers. Out of 154 hypertensive mothers, 29.87% had primary education while the total number of normotensive mothers who had primary education was 21.43%. Also, total hypertensive mothers who had secondary education were 25.97%, compared to 42.21% of normotensive mothers who had secondary education (Table 1).

**Table 1:** Socio-demographic characteristics of the newborns and mothers at University of Gondar comprehensive specialized hospital from March to May 2022

Variables	Categories	Case group N (%)	Control group N (%)
Newborns’ sex	Male	64 (41.56)	70 (45.45)
	Female	90 (58.44)	84 (54.55)
Mothers’ age	< 20 years	4 (2.60)	0(0)
	20–34 years	129 (83.77)	140 (90.91)
	35–39 years	18 (11.69)	12 (7.79)
	≥ 40 years	3 (1.95)	2 (1.30)
Mothers’ residence	Urban	94 (61.04)	112 (72.73)
	Rural	60 (38.96)	42 (27.27)
Mother educational status	No formal education	29 (18.83)	19 (12.34)
	Primary school	46 (29.87)	33 (21.43)
	Secondary school	40 (25.97)	65 (42.21)
	High school and above	39 (25.32)	37 (24.02)
Mother occupational status	Housewife	80 (51.95)	70 (45.45)
	Merchant	17 (11.04)	25 (16.23)
	Private employee	33 (21.43)	44 (28.57)
	Governmental employee	21 (13.64)	13 (8.44)
	Students	3 (1.95)	2 (1.30)

### Obstetrics and nutritional characteristics of newborns and mothers

Around 77% of HDP and 84% of normotensive mothers had taken iron or vitamin supplements. There was no history of abortion in 84% and 88% of hypertensive and normotensive mothers respectively. The mean gestational age of delivery in hypertensive mothers was 36.75 ± 2.30 weeks, while in normotensive mothers was 38.79 ± 1.62 weeks (P = 0.000). The need for resuscitation was around 42% in cases and around 16% in controls (Table 2).

**Table 2:** The obstetric and nutritional characteristics of the newborns and mothers at University of Gondar comprehensive specialized hospital from March to May 2022

Variables	Category	Case group	Control group
		N (%)	N (%)
Gravity	Primigravida	60 (38.96)	70 (45.45)
	Multigravida	94 (61.04)	84 (54.55)
Parity	1	63 (40.91)	72 (46.75)
	2	41 (26.62)	48 (31.17)
	3	28 (18.18)	15(9.74)
	4	13 (8.44)	15 (9.74)
	≥ 5	9 (5.85)	4 (2.60)
Birth interval in years	< 3 years	40 (43.95)	27 (32.93)
	3-5 years	48 (52.75)	50 (60.98)
	> 5 years	3 (3.30)	5 (6.09)
History of abortion	Yes	24 (15.58)	17 (11.04)
	No	130 (84.42)	137 (88.96)
Vegetable consumption per week	Never	7 (4.55)	13 (8.44)
	Once a week	5 (3.25)	1 (0.65)
	2–3 days a week	47 (30.52)	34 (22.08)
	Above 3 days	95 (61.69)	106 (68.83)
Fruit consumption per week	Never	14 (9.09)	24 (15.58)
	Once a week	16 (10.39)	3 (1.95)
	2–3 days a week	78 (50.65)	62 (40.26)
	Above 3 days	46 (29.87)	65 (42.20)
ANC follow up	Yes	127 (82.47)	136 (88.31)
	No	27 (17.53)	18 (11.69)
Take iron/ vitamin	Yes	119 (77.27)	129 (83.77)
	No	35 (22.73)	25 (16.23)
Gestational maturity	Pre term	74 (48.05)	19 (12.34)
	Term	80 (51.95)	135 (87.66)
Mode of delivery	SVD	93 (60.39)	113 (77.38)
	Induced	4 (2.60)	3 (1.95)
	Assisted	5 (3.25)	4 (2.60)
	CS	55 (33.77)	34 (22.08)
Resuscitation for newborns	Yes	66 (42.86)	25 (16.23)
	No	88 (57.14)	129 (83.77)

*Note*: ANC: Anti natal care, CS: Caesarians section, SVD: Spontaneous vaginal delivery

### The anthropometric measurements of the newborns and mothers

The mean BMI of HDP mothers was 24.24 ± 2.51 kg/meter^2^, while that of normotensive mothers was 24.25 ± 2.28 kg/meter^2^ (P = 0.974). The mean weights of cases were 2642.20 ± 496.10 g, while in controls were 2944.80 ± 426.26 g (P = 0.000) (Table 3).

**Table 3:** The anthropometric and blood pressure measurements of the newborns and mothers at University of Gondar comprehensive specialized hospital from March to May 2022

Variables	Case group	Control group	P- value
	Mean ± SD	Mean ± SD	
BMI of mothers in grams	24.24 ± 2.51	24.25 ± 2.28	0.974
SBP of mothers in mmHg	166.17 ± 13.15	110.48 ± 5.96	0.000*
DBP of mothers mmHg	111.97 ± 9.53	70.06 ± 5.97	0.000*
Weight of newborn in grams	2642.20 ± 496.10	2944.80 ± 426.26	0.000*

*Note*: SD: Standard deviation, * indicates p-value < 0.05 statistically significant

### Hematological profiles of newborns

The mean TLC of cases was 11.42 ± 4.26 × 10^3^/ul, while in the control group it was 12.48 ± 4.41 × 10^3^/ul. The mean ANC of cases was 6.62 ± 3.30 × 10^3^/ul, while in controls it was 7.55 ± 3.31 × 10^3^/ul. The TLC (P = 0.015); ANC and RBC (P = 0.007),;monocyte (P = 0.026), (P < 0.001); IRF (P = 0.020); MRV (P = 0.002; PLT counts, the Hgb, hematocrit, MCV, MCH, RDW and nRBC (P < 0.001) were significant difference between cases and controls (Table 4).

**Table 4:** Hematological profile of newborns from hypertensive and normotensive mothers delivered at the University of Gondar comprehensive specialized hospital from March to May 2022

Hematological parameters	Case group	Control group	P-Value
TLC (10*3/µL)^a^	11.42 ± 4.26	12.48 ± 4.41	0.015*
ANC (10*3/µL) ^a^	6.62 ± 3.30	7.55 ± 3.31	0.007*
RBC (10*6/µL)^a^	4.41 ± 0.40	4.26 ± 0.64	0.007*
Hematocrit (%) ^a^	49.10 ± 5.19	46.09 ± 7.63	< 0.001*
MCV (FL)^a^	110.21 ± 7.48	107.80 ± 5.97	< 0.001*
MCHC (g/dl)^a^	34.20 ± 1.98	34.01 ± 1.28	0.473
RDW-SD (FL)^a^	71.97 ± 10.16	68.79 ± 8.88	0.001*
NRBC/100 WBC^a^	7.41 ± 3.46	4.68 ± 2.33	< 0.001*
MRV(FL)^a^	143.61 ± 9.42	140.40 ± 10.53	0.002*
Platelet (10*3/µL) ^a^	221.25 ± 83.56	260.24 ± 83.01	< 0.001*
ALC (10*3/µL) ^b^	3.1 (2.2, 4)	3.1 (2.4, 4.1)	0.468
Monocyte count (10*3/µL) ^b^	3.1 (2.2, 4)	1.1 (0.8, 1.5)	0.026*
Eosinophil count (10*3/µL)^b^	0.2 (0.1, 0.3)	0.2 (0.1, 0.4)	0.354
Basophil count (10*3/µL)^b^	0.1 (0.1, 0.2)	0.1 (0.1, 0.2)	0.527
Hgb (g/dL)^b^	16.7 (15.8, 17.5)	16.05 (15, 17.2)	< 0.001*
MCH (pg) ^b^	37.9 (36.5, 38.5)	36.95 (35.7, 37.9)	< 0.001*
RDW (%) ^b^	18.55 (17.5, 19.5)	17.7 (16.9, 18.7)	< 0.001*
MPV (FL)^b^	8.2 (7.8, 8.7)	8.1 (7.6, 8.6)	0.160
Reticulocyte (%) ^b^	3.33 (2.97, 3.92)	3.29 (2.83, 3.74)	0.171
Reticulocyte (FL) ^b^	0.14 (0.12, 0.18)	0.13 (0.12, 0.16)	0.080
IRF ^b^	0.63 (0.60, 0.66)	0.64 (0.61, 0.67)	0.020*

*Note*: TLC: Total leucocyte count, ANC: Absolute neutrophil count, ALC: Absolute lymphocyte count, RBC: Red blood cell, Hgb: Hemoglobin, MCV: Mean cell volume, MCHC: Mean cell hemoglobin concentration, MCH: Mean cell hemoglobin, RDW: Red cell distribution width, nRBC: Nucleated red blood cells, WBC: White blood cells, MRV: Mean reticulocyte volume, MPV: Mean platelet volume, IRF: immature reticulocyte fraction, FL: Femto litters

**NB**: ^a^Values are given as mean ± SD, ^b^Values are given as median (IQR), *indicates statistically significant at p-value < 0.05

**Fig. 1:**
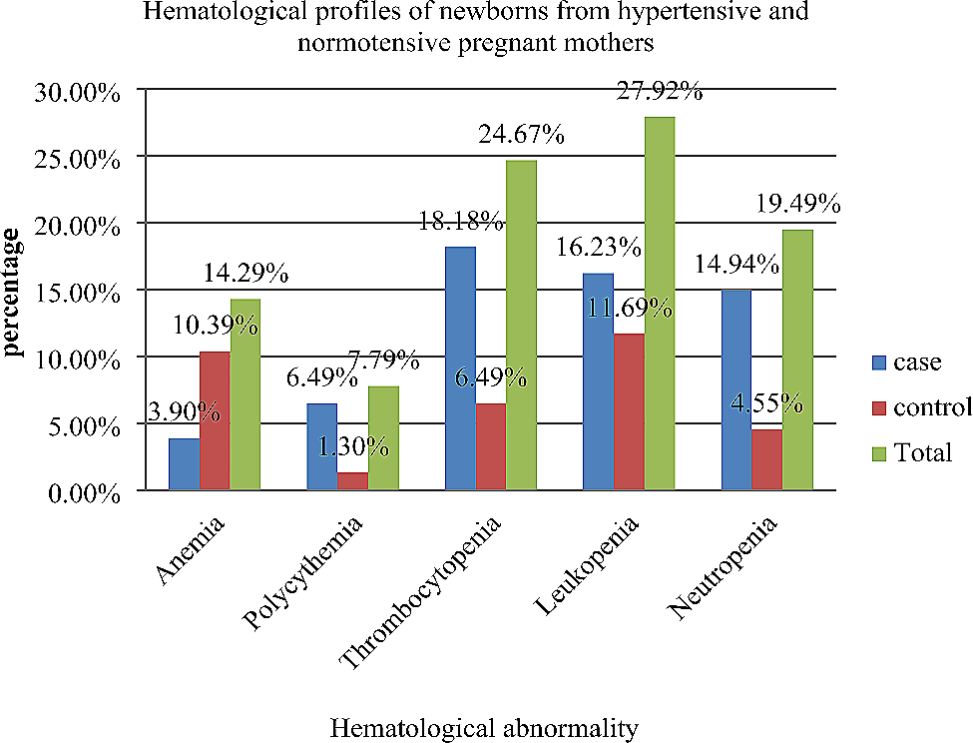
Hematological abnormalities among newborns from hypertensive and normotensive pregnant mothers attending at the University of Gondar Comprehensive Specialized Hospital, 2022

In the current study, the overall prevalence of thrombocytopenia, leukopenia, and neutropenia among cases was 18.18% (28/154) (95% CI: 12.80, 25.16), 16.23% (25/154) (95% CI: 11.16, 23.0), and 14.94% (23/154) (95% CI: 10.08, 21.55), respectively. However, in the control group, the prevalence of thrombocytopenia, leukopenia, and neutropenia was 6.49% (10/154) (95% CI: 3.50, 11.72), 11.69% (18/154) (95% CI: 7.45, 17.87) and 4.55 (7/154) (95% CI: 2.16, 9. 29), respectively (Fig. 1).

### Hematological profiles of newborns from different types of HDP

The mean platelet count in newborns from mothers with superimposed preeclampsia/eclampsia was 176 ± 89 × 10^3^/ul while it was 266 ± 64 × 10^3^/ul in newborns from gestational hypertension mothers (P < 0.001). The mean of nRBC was 9 ± 4/100 WBC and 6 ± 2 /100WBC in newborns from mothers with superimposed preeclampsia/eclampsia and gestational hypertension respectively (P < 0.001) (Table 5).

**Table 5:** Hematological profile of the newborn from mothers with different types of HDP delivered at university of Gondar Comprehensive specialized hospital from March to May 2022

Hematological parameters	Gestational hypertension (n = 39)	Preeclampsia/ eclampsia (n = 39)	Chronic hypertension (n = 38)		Superimposed Preeclampsia (n = 38)	P-value
TLC (10*3/µL)^a^	12.3 ± 3.6	12.1 ± 3.1	10.8 ± 5.2	10.5 ± 4.8		0.179
ANC (10*3/µL) ^a^	7.2 ± 2.9	6.9 ± 2.2	6.7 ± 3.9	5.7 ± 3.9		0.250
RBC (10*6/µL)^a^	4.36 ± 0.45	4.37 ± 0.45)	4.45 ± 0.36	4.48 ± 0.35		0.448
Hematocrit (%) ^a^	48.4 ± 6.8	49.0 ± 3.5	49.3 ± 4.1	49.7 ± 5.9		0.756
MCV (FL)^a^	109.4 ± 7.4	110.3 ± 4.4	110.4 ± 5.5	110.7 ± 11.2		0.888
MCHC (g/dl)^a^	33.8 ± 1.0	33.9 ± 1.2	34.2 ± 1.4	34.3 ± 1.5		0.473
RDW-SD (FL)^a^	70.6 ± 11.7	71.6 ± 8.4	72.8 ± 8.8	72.9 ± 11.6		0.754
NRBC/100 WBC^a^	6 ± 2	6 ± 2	8 ± 3	9 ± 4		< 0.001*
MRV(FL)^a^	144.9 ± 10.2	144.7 ± 10.6	141.6 ± 9.0	143.3 ± 7.6		0.391
Platelet (10*3/µL) ^a^	266 ± 64	258 ± 50	182 ± 84	176 ± 89		< 0.001*
ALC (10*3/µL) ^b^	3.1 (2.6, 4.4)	3.4 (2.6, 4)	2.85 (1.5, 3.6)	2.65 (2.2, 3.8)		0.182
Monocyte (10*3/µL) ^b^	1.1 (0.7, 1.4)	1 (0.7, 1.4)	0.9 (0.7, 1.1)	0.9 (0.7,1.2)		0.456
Eosinophil (10*3/µL)^b^	0.3 (0.2, 0.3)	0.2 (0.1, 0.4)	0.1 (0.1, 0.3)	0.2 (0.1,0.3)		0.188
Basophil (10*3/µL)^b^	0.2 (0.1, 0.2)	0.1 (0.1, 0.2)	0 0.1 (0.1, 0.2)	0.1(0.1,0.2)		0.511
Hgb (g/dL)^b^	16.5 (15.7, 17.5)	16.7 (15.9, 17.5)	16.75 (14.8, 17.5)	17.15 (15.9,17.7)		0.681
MCH (pg) ^b^	37.5 (36.4, 38.4)	37.7 (36.1, 38.4)	37.9 (36.4, 38.5)	38.25 (37.7,38.8)		0.145
RDW (%) ^b^	17.8 (17.3, 19.3)	18.5 (17.8, 19.5)	18.7 (17.2, 19.6)	18.9 (17.6, 19.4)		0.454
MPV (FL)^b^	8.4 (8.0, 9.2)	8.3 (7.6, 8.6)	8.2 (7.8,8.7)	8.1 (7.8, 8.4)		0.291
Reticulocyte (%) ^b^	3.42 (3.01, 3.95)	3.31 (2.85, 3.99)	3.16 (2.86, 3.91)	3.3(3.05, 3.92)		0.577
Reticulocyte (FL) ^b^	0.15 (0.13,0.18)	0.15 (0.12,0.18)	0.14 (0.12, 0.17)	0.14 (0.12,0.17)		0.619
IRF ^b^	0.63 (0.61, 0.65)	0.65 (0.59, 0.67)	0.63 (0.61, 0.65)	0.63 (0.60, 0.65)		0.983

*Note*: TLC: Total leucocyte count, ANC: Absolute neutrophil count, ALC: Absolute lymphocyte count, RBC: Red blood cell, Hgb: Hemoglobin, MCV: Mean cell volume, MCHC: Mean cell hemoglobin concentration, MCH: Mean cell hemoglobin, RDW: Red cell distribution width, nRBC: Nucleated red blood cells, WBC: White blood cells, MRV: Mean reticulocyte volume, MPV: Mean platelet volume, IRF: immature reticulocyte fraction, FL: Femto litters

**NB**: ^a^ Values are given as mean ± SD, ^b^ Values are given as median (IQR), * indicates statistically significant at p-value < 0.05

Post hoc test analysis showed that platelet counts were statistically different between newborns from mothers with gestational hypertension and those with chronic hypertension (P < 0.001), and there was a significant difference between superimposed preeclampsia/eclampsia and those with preeclampsia/eclampsia (P < 0.001). The nRBC showed a significant difference between newborns with superimposed preeclampsia/eclampsia and gestational hypertension (P < 0.001), as well as a significant difference between newborns from mothers with superimposed preeclampsia/eclampsia and preeclampsia/eclampsia (P < 0.001) (Table 6).

**Table 6:** Post hoc test for platelet count and nRBC/ 100 WBC of newborns from mothers with different types of HDP delivered at university of Gondar Comprehensive specialized hospital from March to May 2022

Hematological parameters	Types of HDP	Gestational hypertension (P-value)	Preeclampsia/ eclampsia (P-value)	Chronic hypertension (P-value)
Platelet	Gestational hypertension	1.000	1.000	< 0.001*
	Preeclampsia/ ecalmpsia	1.000	1.000	< 0.001*
	Superimposed Preeclampsia	< 0.001*	< 0.001*	1.000
NRBC/ 100 WBC	Gestational hypertension	1.000	1.000	0.019*
	Preeclampsia/ eclampsia	1.000	1.000	0.003*
	Superimposed Preeclampsia	< 0.001*	< 0.001*	1.000

*Note*: HDP: Hypertensive disorder of pregnancy, nRBC: Nucleated red blood cells, WBC: White blood cells

**NB**: * indicates statistically significant at p-value < 0.05

### The correlation of Independent variables with hematological profiles of cases

The TLC of the case found a positive correlation between gestational age (P = 0.009) and newborn weight (P = 0.025). TLC of cases showed a statistically negative correlation with DBP (P = 0.042) and SBP of mothers (P = 0.002). The ANC of cases was found to be positively correlated with gestational age (P = 0.001) and newborn weight (P = 0.007). The ANC of cases, on the other hand, was inversely correlated with DBP (P = 0.020) and SBP of mothers (P < 0.001). The monocyte counts of cases were found to be positively correlated with gestational age (P = 0.040) and newborn weight (P < 0.001). In addition, the eosinophil and basophil count of cases were found to be significantly positive correlated with the weight of newborns (P = 0.043 and P = 0.001 respectively).

The platelet counts of cases were found to be positively correlated with gestational age (P-value < 0.001) and newborn weight (P-value < 0.001). Also, the platelet counts of cases were found to be positively correlated with the BMI of mothers (P-value < 0.001). The MCHC of cases had a positive correlation with newborn weight (P-value = 0.011). The nRBC of cases was found to be negatively correlated with the BMI of mothers (P-value = 0.011), gestational age of delivery (P-value < 0.001), and newborn weight (P-value = 0.001) and positively correlated with DBP (P-value = 0.033) and SBP of mothers (P-value = 0.007). The TLC and platelet counts were negative correlations with the duration of hypertension (P-value = 0.033) (P-value = 0.001) respectively, while, the nRBC were positively correlated with the duration of hypertension (P-value = 0.024) (Table 7).

**Table 7:** The correlation of independent variables with hematological parameters of newborns from hypertensive mothers at the university of Gondar comprehensive specialized hospital from March to May2022

Hematological parameters	BMI (P-value)	DBP(P-value)	SBP (P-value)	Gestational age (p-value)	Newborn’s weight (p-value)	Duration of HDP (P-value)
WBC ^r^	0.154 (0.055)	-0.164 (0.042*)	-0.247 (0.002*)	0.2082 (0.009*)	0.1804 (0.025*)	-0.1715 (0.033*)
ANC ^r^	0.2063 (0.010*)	-0.1868 (0.020*)	-0.2690 (< 0.001*)	0.2634 (0.001*)	0.214 (0.007*)	-0.0698 (0.389)
RBC count ^r^	-0.0394 (0.627)	-0.1216 (0.133)	-0.0178 (0.826)	0.0444 (0.584)	0.0728 (0.369)	-0.0732 (0.366)
Hematocrit ^r^	-0.0640 (0.340)	-0.0679 (0.402)	0.0033 (0.967)	-0.0927 (0.252)	0.0560 (0.490)	-0.0432 (0.595)
MCV ^r^	-0.0458 (0.572)	0.0526 (0.517)	0.0973 (0.229)	-0.1535 (0.057)	-0.0868 (0.284)	-0.0351 (0.665)
MCHC ^r^	0.0284 (0.726)	0.0005 (0.995)	0.0191 (0.814)	0.2029 (0.011*)	-0.0114 (0.888)	0.0826 (0.308)
RDW-SD ^r^	-0.0336 (0.678)	0.0357 (0.660)	0.0355 (0.662)	-0.1958 (0.014)	-0.0218 (0.788)	-0.0511 (0.529)
NRBC ^r^	-0.2026 (0.011*)	0.1712 (0.033*)	0.2149 (0.007*)	-0.3252 (< 0.001*)	-0.2582 (0.001*)	0.1812 (0.024*)
MRV ^r^	0.0455 (0.575)	0.0259 (0.749)	-0.0508 (0.531)	0.0316 (0.697)	0.0701 (0.387)	-0.1018 (0.209)
Platelet ^r^	0.4446 (< 0.001*)	-0.3222 (< 0.001*)	-0.2751 (< 0.001*)	0.3118 (< 0.001*)	0.4517(< 0.001*)	-0.2606 (0.001*)
MPV ^r^	-0.0990 (0.221)	0.1848 (0.021*)	0.1257 (0.120)	-0.1878 (0.019)	-0.0455 (0.575)	0.1965 (0.014*)
ALC ^rho^	-0.0174 (0.830)	-0.0283 (0.727)	-0.0625 (0.441)	0.0520 (0.521)	0.0821 (0.311)	-0.1230 (0.128)
Monocyte # ^rho^	0.2186 (0.006*)	-0.2836 (< 0.001*)	-0.3231 (0.001*)	0.1655 (0.040*)	0.302 (< 0.001*)	-0.1813 (0.024*)
Eosinophil# ^rho^	0.1857 (0.021*)	-0.2419 (0.002*)	-0.1193 (0.014*)	0.0770 (0.342)	0.1632 (0.043*)	-0.0775 (0.339)
Basophil # ^rho^	0.0988 (0.223)	-0.2711 (< 0.001*)	-0.2792 (0.001*)	0.0839 (0.300)	0.2578 (0.001*)	-0.0712 (0.380)
Hgb ^rho^	-0.0911 (0.261)	-0.1058 (0.191)	0.0529 (0.514)	-0.0223 (0.784)	0.0348 (0.668)	-0.1210 (0.134)
MCH ^rho^	0.0012 (0.988)	0.0664 (0.413)	0.0414 (0.609)	-0.0421 (0.604)	-0.0152 (0.851)	-0.0162 (0.842)
RDW ^rho^	0.0146 (0.857)	0.0822 (0.310)	0.0180 (0.824)	-0.0862 (0.287)	0.0164 (0.840)	-0.0440 (0.588)
Retic % ^rho^	0.1121 (0.166)	0.0008 (0.992)	-0.0147 (0.856)	-0.0143 (0.860)	-0.0145 (0.858)	-0.0509 (0.530)
Retic #^rho^	0.0909 (0.262)	-0.0796 (0.326)	-0.0327 (0.687)	-0.0146 (0.857)	0.0091 (0.910)	-0.1027 (0.204)
IRF ^rho^	0.0317 (0.696)	0.0113 (0.889)	0.0317 (0.695)	0.0346 (0.669)	0.0215 (0.790)	0.0749 (0.355)

*Note*: TLC: Total leucocyte count, ANC: Absolute neutrophil count, ALC: Absolute lymphocyte count, RBC: Red blood cell, Hgb: Hemoglobin, MCV: Mean cell volume, MCHC: Mean cell hemoglobin concentration, MCH: Mean cell hemoglobin, RDW: Red cell distribution width, MPV: Mean platelet volume, retic: reticulocyte, nRBC: Nucleated red blood cells, WBC: White blood cells, MRV: Mean reticulocyte volume, IRF: immature reticulocyte fraction, FL: Femto litters

**NB**: * indicates statistically significant at p-value < 0.05. r = Pearson correlation, rho = Spearman’s rank correlation, # = absolute count

## Discussion

Hypertensive disorders in pregnancy result in a decline in prenatal placental perfusion and insufficient blood supply to the fetus, resulting in fetal exposure to hypoxia or oxidative stress in the placenta. The hypoxic placenta releases particular vasoactive substances into neonatal blood in response to this stress, which affects the hematological profiles of newborns [8, 9].

In the current study, the mean gestational age of delivery in hypertensive mothers was lower than in normotensive mothers. The mean weights of cases were lower than controls. The probable reason is that hypertension causes uteroplacental failure and hypoxia, and the hypoxia leads to low birth weight [15]. Another reason may be due to preterm delivery or intrauterine growth restriction causing low birth weight in newborns [35].

In the present study, the mean TLC was lower in cases compared to controls. The prevalence of leukopenia among cases was 16.23%. However, in the control group, it was 11.69%. The current study’s findings are similar to those of studies conducted in Sudan [36], Egypt [37], Tanzania [20], Turkey [15], Iraq [38], and India [39]. The possible mechanism of the HDP’s lowering TLC may be the resulting uteroplacental insufficiency that causes inhibited fetal myeloid bone marrow production [15].

Furthermore, the mean ANC was lower in cases compared to controls in the current study. Also, the overall prevalence of neutropenia among cases was significantly higher compared to controls. The findings of the present study were in agreement with studies done in Nigeria [11], Tanzania [20], Korea [40], Turkey [15], and India [39]. One potential mechanism is that HDP and the resultant uteroplacental insufficiency inhibit fetal bone marrow production of the myeloid lineage, manifested by a decrease in neutrophil production [15]. Additionally, ANC reduction had other possible mechanisms. It could be due to the interaction of Fas-to-Fas ligand for apoptosis pathway activation, which causes the increase of Fas-associated apoptosis protein in the mother’s and newborn’s blood. The increased apoptotic activity of myeloid precursors may contribute to the reduction of ANC in newborns [41, 42]. Another possible mechanism may be due to the increase of placental-derived inhibitors of neutrophil formation, which suppress natural G-CSF production. As a result, the ANC was reduced in newborns. Reduced levels of circulating CFU-GM and neutrophil storage pools are also linked to ANC reduction [43].

In the present study, the median monocyte counts were lower in cases compared to controls. The possible mechanism of the HDP’s lowering of monocyte counts may be due to the resulting uteroplacental insufficiency that prevented fetal myeloid bone marrow production [15]. This study was in agreement with the study conducted in Turkey [15]. However, this result is not similar to the study conducted in Nigeria [44], which reported that monocyte counts were higher in cases than controls. The possible variation of monocyte counts in this study compared to other studies may be due to variations in nutrition, genetics, environmental factors, and the sample size of the study.

In the current study, cases had significantly higher mean RBC counts compared to controls. This could be explained by the relative hypoxia the fetus experiences during a pregnancy, exacerbated by hypertension, which prompts the production of erythropoietin, which in turn encourages erythropoiesis, resulting in an increase in RBC counts in neonates [8, 9]. Similar studies conducted in Egypt [37] and India [45, 46] reported that the RBC count was significantly higher in cases than controls. However, the findings of the study were in contrast to a study conducted in Tanzania [20], which showed that the median RBC counts were significantly lower in cases compared to controls. The RBC variations in the study might be due to variations in nutrition, genetics, environmental factors, and sample size.

The present study showed that the median Hgb levels were significantly higher in cases compared to controls. In the current study, the prevalence of anemia among cases was 3.90%. However, in controls, it was 10.39%. The mechanism of increased Hgb in the cases may be due to HDP causing uteroplacental insufficiency, which leads to fetal hypoxia and stimulates erythropoiesis. For this reason, Hgb levels were increased in newborns of hypertensive mothers [8, 9]. Another reason may be that newborns delivered from hypertensive mothers have higher amounts of Hgb F than newborns from normotensive mothers [15]. As a result, HgbF has a greater affinity for oxygen than HgbA. This is because HbF does not interact with 2,3-diphosphoglycerate in a significant manner, and cells that have HbF have a higher oxygen affinity and the benefit of drawing more oxygen from the mother’s blood through the placenta. This leads to uteroplacental failure, and hypoxia. The findings were consistent with the previous studies conducted in Egypt [37], Nigeria [44], Indonesia [47], and India [46, 48]. Whereas the findings were in contrast to studies conducted in Sudan [36] and India [45], which showed that Hgb was lower in cases than controls. The Hgb variations in the study might be due to variations in nutrition, genetics, environmental factors, and sample size.

In the current study, the mean MCV and MCH were significantly higher in cases compared to controls. The study is comparable to those conducted in Sudan [36] and India [46]. In the present study, the mean nRBC/100 WBC was higher in cases than controls. The findings of to those of study were similar to studies conducted in India [39, 45, 46, 49–51]. The possible mechanisms of HDP to increase MCV, MCH, and nRBC/100 WBC are a decline in prenatal placental perfusion with insufficient blood supply to the fetus, resulting in fetal exposure to hypoxia or oxidative stress in the placenta [8, 9]. Reduced oxygen tension activates compensatory mechanisms that increase immature RBCs. The MCV, MCH, and nRBC/100 WBC were increased [10, 11]. The cases had a higher median value of RDW compared to the controls. The possible mechanism of HDP to increase RDW is a decline in prenatal placental perfusion with insufficient blood supply to the fetus, resulting in fetal exposure to hypoxia or oxidative stress in the placenta [8, 9]. Reduced oxygen tension triggers compensatory mechanisms that increase immature RBCs. Therefore, there is a variation in the size of the RBC. As a result, the RDW is increased in newborns from HDP mothers [10, 11]. The findings of this study are in agreement with studies conducted in Sudan [36], Tanzania [20], and India [19].

The mean platelet count of the cases was lower than that of the controls. Also, the overall prevalence of thrombocytopenia among cases was significantly higher than in control groups. This study is similar to studies conducted in Egypt [37], Nigeria [11], Tanzania [20], Iraq [38], Korea [40], and India [39, 48, 50, 51]. Fetal hypoxia, which directly inhibits fetal megakaryocytopoiesis and platelet production, is one potential cause for the decrease in platelet count [11]. The most likely causal mechanism is assumed to be a combination of defective megakaryocyte development and enhanced platelet activation mediated by cytokines, thrombopoietin, and interleukin-6. The platelet count reduction can also be caused by thrombocyte adhesion to the injured endothelium area in hypertensive women’s placenta, which is triggered by segmental vasospasm and vasodilation [52–54].

The current study showed that the mean platelet counts of newborns were statistically significantly different between different types of HDP mothers. The present study showed that newborns from superimposed preeclampsia/eclampsia mothers had lower mean platelet counts than those with chronic hypertension, gestational hypertension, and preeclampsia/eclampsia mothers. There was also a statistically significant difference between newborns from gestational hypertension and chronic hypertension mothers, between preeclampsia/eclampsia and chronic hypertension, between superimposed preeclampsia/eclampsia and gestational hypertension mothers, and between superimposed preeclampsia/eclampsia and Preeclampsia/eclampsia. The finding of the study was comparable to studies conducted in India, found that platelet counts were a statistically significant difference between newborns with different types of HDP [19, 55]. The reason could be that the severity and duration of hypertension are important in influencing the platelet counts of neonates. Due to increasing the fetus’s exposure to hypertension, which greatly impacts the platelet count of newborns [19].

The current finding revealed that the mean nRBC/100 WBC in newborns with superimposed preeclampsia/eclampsia was higher compared to newborns with chronic hypertension, gestational hypertension and preeclampsia/eclampsia mothers. Besides, nRBC/100 WBC showed a statistically significant difference between newborns from gestational hypertension and chronic hypertension, between superimposed preeclampsia and gestational hypertension, and between superimposed preeclampsia and preeclampsia. The increase in nRBC among newborns from superimposed preeclampsia/eclampsia is most likely due to chronic placental hypoxia, which raises erythropoietin levels, leading to activated erythropoiesis in newborns [10, 11].

In the present study, TLC, ANC, monocyte count, and platelet counts of cases found a statistically significant positive correlation with the gestational age and the weight of the newborn. The findings of the current study were similar to those conducted in Romania [56]. The possible reason may be that maternal hypertension has a direct impact on the development of hematological abnormalities in newborns, whereas premature delivery and weight of newborns are additional aggravating factors for TLC, ANC, monocyte count, and platelet counts in newborns [18]. Also, TLC, ANC, monocyte, eosinophil, basophil, and platelet counts in cases showed a significant negative correlation between DBP and SBP of mothers. The findings of study are similar to those conducted in Romania and India [55, 56]. The possible reason could be increasing the fetus’s exposure to high blood pressure, which has a great impact on TLC, ANC, monocyte, eosinophil, basophil, and platelet counts of newborns [19].

In this study, the eosinophil and basophil count of cases were found to be significantly positive correlated with the weight of newborns. The possible reason may be that maternal hypertension directly affects the hematological profiles of newborns, whereas the weight of the newborns is an additional aggravating factor for eosinophil and basophil counts of newborns [18]. Additionally, the eosinophil counts of cases were found to be significantly positively correlated with the BMI of the mother. The probable reason could be the fact that adipose tissue is a great source of inflammatory factors such as interleukin (IL)-6 and IL8, which are also important inducers of eosinophil production. As a result, the BMI of the mother was reduced, which caused the reduction of eosinophil production in newborns [57].

The nRBC of cases was found to be statistically significantly negatively correlated with gestational age and newborn weight and significantly positively correlated with the DBP and SBP of mothers. The possible justification may be due to maternal hypertension having a direct effect on the increase of nRBCs in newborns, whereas premature delivery and weight of newborns are additional aggravating factors for nRBCs in newborns [18]. The possible reason of the increased blood pressure of mothers causing the increase of nRBCs in newborns is due to the increasing the fetus’s exposure to hypertension, which has a great influence on the nRBCs of newborns [19]. The current study showed that TLC and PLT counts were negatively correlated with the duration of hypertension while they were positively correlated with nRBC. The reason for the decrease of PLT and TLC, and the increase of nRBC may be due to the duration of hypertension because of increasing the fetus’s exposure to hypertension for a long time, which has a great impact on the PLT, TLC, and nRBC counts of newborns [19].

### Limitation of the study

The limitation of this study was that the hematological profiles of mothers were not determined, the infants were not followed up to investigate the effects of HDP in the neonatal period, and the study did not consider the percentage of small gestational age neonates in each group, which may play a role in hematological parameters. Additionally, the clamping times of the cord were not assessed.

## Conclusions

Most of WBC parameters, platelet counts, and IRF were significantly lower in cases compared to controls. Majority of RBC parameters were significantly higher in cases compared to the controls. The mean platelet counts and nRBC had significant differences between newborns from gestational hypertension and chronic hypertension, between superimposed preeclampsia and gestational hypertension, and between superimposed preeclampsia and preeclampsia mothers. The white blood cell parameters and platelet counts in cases had a statistically significant positive correlation with gestational ages of delivery and weights of the newborn. However, the white blood cell parameters and platelet counts in cases showed a statistically significant negative correlation between DBP and SBP of mothers. The nRBC among cases was found to have significant negative correlation with gestational age and newborn weight, and statistically significant positive correlated with DBP and SBP of mothers.

Early hematological screening of newborns from HDP mothers using laboratory tests such as CBC aids in the early detection of hematological complications. Furthermore, early hematological screening of newborns from HDP mothers is recommended to aid in the early detection and management of hematological abnormalities to reducing infection and bleeding complications. We recommend researchers assess the hematological profiles of mothers; infants to be followed up to investigate the long-term effects of hypertensive disorder in the neonatal period; should consider the percentage of small gestational age neonates in each group and clumping times of cord blood for a similar study.

## Data Availability

The data are available from the corresponding author upon reasonable request.

## Abbreviations

ALC: Absolute Lymphocyte Count
ANC: Absolute Neutrophil Count
BMI: Body Mass Index
CBC: Complete Blood Count
CFU-GM: Colony-Forming Unit-Granulocyte Macrophage
DBP: Diastolic Blood Pressure
G-CSF: Granulocyte Colony Stimulating Factor
HDP: Hypertensive Disorders of Pregnancy
Hb: Hemoglobin
IRF: Immature Reticulocyte Fraction
MCH: Mean Cell Hemoglobin
MCHC: Mean Cell Hemoglobin Concentration
MCV: Mean Cell Volume
MPV: Mean Platelet Volume
MRV: Mean Reticulocyte Volume
NRBC: Nucleated Red Blood Cell
RBC: Red Blood Cell
RDW: Red cell Distribution Width
SBP: Systolic Blood Pressure
SD: Standard Deviation
TLC: Total Leucocyte Count
WBC: White Blood Cell
